# Maintenance Therapies for Non-Small Cell Lung Cancer

**DOI:** 10.3389/fonc.2014.00213

**Published:** 2014-08-19

**Authors:** Normand Blais, Elie Kassouf

**Affiliations:** ^1^Medical Oncology, Centre Hospitalier de l’Université de Montréal, Montreal, QC, Canada

**Keywords:** maintenance, pemetrexed, erlotinib, gefitinib, bevacizumab, lung cancer, docetaxel

## Abstract

Treatment of lung cancer had evolved during the last decade with the introduction of new chemotherapeutic regimens and targeted therapies. However, the maximum benefit reached after first-line therapy is limited by the cumulative toxicity of platinum drugs and the subsequent deterioration in performance status in a high percentage of patients who end up receiving not more than one line of treatment. Maintenance therapy had been introduced and evaluated in many large randomized trials showing a delay in tumor progression and an improvement in overall survival. This effective strategy should be taken into account when discussing the initial treatment plan and tailored according to the preferences of both patients and physicians.

## Introduction

Standard first-line treatment for patients who are negative for the EGFR mutation and ALK rearrangement consists of platinum-based chemotherapy (1). The optimal number of cycles had been determined after Park et al. compared four to six cycles of treatment showing non-inferiority in terms of overall survival and thus, making four cycles a currently accepted standard (2).

During the past decade, research from different work groups has focused on finding alternative strategies to improve tumor response and extend survival.

The limited benefit from extending platinum-based chemotherapy beyond four cycles as well as the cumulative toxicities of these regimens leading to worsening of the quality of life (QoL) (2–4) had led to the re-emergence of a relatively new concept based on maintaining the response in patients who attain tumor control during first-line induction treatment.

Therefore, cytotoxic agents and molecularly targeted agents have been extensively evaluated in this setting and two practical applications of maintenance therapy have evolved: continuation maintenance and switch maintenance.

Maintenance therapy has potential advantages and inconveniences. Although many large randomized studies have shown that maintenance therapy is associated with a delay in tumor progression and an improvement in overall survival, prolonging therapy in a palliative intent also significantly increases the burden of medical interventions to a patient, prohibits the patient from having an often desired treatment holiday and may also increase treatment related toxicities, which may have a detrimental impact on QoL.

This mini review will expose the issues related to maintenance therapy and discuss a personalized approach to the implementation of such strategies in clinical practice.

## Continuation Maintenance

Continuation maintenance refers to the continuation of one or more non-platinum agents, initially used during induction, until progression. This approach allows the discontinuation of platinum compounds known to cause cumulative toxicity that often becomes clinically meaningful after four to six doses of these drugs. It also has the advantage of continuing an agent for which tolerance has been defined in the induction phase of the treatment.

In 2006, Sandler et al. published the results of the ECOG 4599 trial, which showed that continuing bevacizumab (Bev) beyond six cycles of paclitaxel (Pac)/carboplatin (Carbo)/Bev added 2 months improvement in OS compared to six cycles of Carbo/Pac alone (HR, 0.79, *p* = 0.003) (5). Similarly, in 2009, Pirker et al. demonstrated in the FLEX study, that maintenance with cetuximab after chemotherapy with cisplatin/vinorelbine/cetuximab significantly improved OS (11.3 vs. 10.1 months; HR, 0.87, *p* = 0.044) compared to cisplatin/vinorelbine alone (6). It is not clear whether the benefit of bevacizumab or cetuximab in these studies is from their integration of the induction phase, the maintenance phase or both. Nonetheless, the design of these trials revived the concept of adapting maintenance as an appropriate clinical strategy.

Gemcitabine was evaluated in three randomized trials. CECOG (7) and IFCT-GFPC 0502 (8) met their primary endpoint showing longer PFS but with no significantly improved OS. These trials show interesting trends toward an increase in overall survival although this interpretation is limited by the small sample size of these trials. In a third trial, Belani et al. failed to demonstrate any advantage of maintenance gemcitabine on PFS and OS. These negative results have been attributed to the fact that most patients (64%) had a poor performance status (PS 2) at randomization in contrast to most other maintenance trials where most of the patients were PS 0 or 1 (9).

More recently, continuation maintenance with pemetrexed (pem) has been evaluated in two large randomized studies. In the PARAMOUNT trial, continuation pem after four cycles of induction with cisplatin/pem demonstrated significant improvement in PFS (4.1 vs. 2.8 months; HR, 0.62; *p* < 0. 0001) and OS (16.9 vs. 14 months; HR, 0.78; *p* = 0.019) (10). In 2009, Patel et al. combined pem and bev as continuous maintenance in a phase II study. Their strategy was safe and resulted in a promising 14.1 months median OS (11). Maintenance with pem and bev was thereafter tested in three major phase III studies. In AVAPERL, there was a 3.6 months improvement in PFS in the Pem/Bev arm compared to the Bev arm, and OS benefit was similar as that reported in PARAMOUNT although the sample size was too small to demonstrate statistical significance (19.8 vs. 15.9 months, HR, 0.88, *p* = 0.32) (12).

Although not a maintenance trial *per se*, PointBreak compared the ECOG 4599 regimen Pac/Carbo/Bev followed by Bev to Pem/Carbo/Bev followed by maintenance with Pem/Bev. Despite the fact that this study did not show significant improvement in OS (median OS, 12.6 vs. 13.4 months; *p* = 0.949) and a slight advantage in PFS favoring the pemetrexed containing regimen (median PFS, 6.0 vs. 5.6 months; HR, 0.83; *p* = 0.012), the study results highlighted different toxicity profiles between the paclitaxel and pemetrexed containing regimens that may lead to a better treatment selection (13). Fatigue and thrombocytopenia were more frequent in the pem arm and neutropenia, neuropathy, and alopecia more frequent in the paclitaxel arm.

The ongoing ECOG 5508 study may better define the optimal choice of maintenance agent after pac/carbo/bev as it randomizes patients for maintenance after induction into three arms: Bev alone, Pem alone, or combined Pem-Bev (Table 1).

**Table 1:** **Key studies addressing continuous maintenance**.

Reference (study name)	*N* (pts)	Maintenance arms	PFS (mo)	HR	*p* Value	OS (mo)	HR	*p* Value
Sandler et al. (5)	850	Bevacizumab	6.2	0.66	<0.001	12.3	0.79	0.003
(ECOG 4599)		Observation	4.5			10.3		
Pirker et al. (6)	850	Cetuximab	4.8	0.943	0.39	11.3	0.871	0.044
(FLEX)		Placebo	4.8			10.1		
Brodowicz et al. (7)	206	Gemcitabine	3.6		<0.001	13		0.195
(CECOG)		BSC	2.0			11		
Perol et al. (8)	464	Gemacitabine	3.8	0.56	<0.001	12.1	0.89	0.3867
(IFCT-GFPC 0502)		Observation	1.9			10.8		
Belani et al. (9)	255	Gemacitabine	3.9		0.58	8.0	0.97	0.84
		Observation	3.8			9.3		
Paz-Ares et al. (10)	539	Pemetrexed	4.1	0.62	<0.001	13.9	0.78	0.0195
(PARAMOUNT)		BSC	2.8			11.0		
Barlesi et al. (12)	253	Pem/Bev	10.2	0.5	<0.001	19.8	0.88	0.32
(AVAPERL)		Bev	6.6			15.9		
Patel et al. (13)	590	Pem/Bev	6.0	0.83	0.012	12.6	1	0.949
(Point break)		Bev	5.6			13.4		

*PFS = progression-free survival; OS = overall survival; BSC = best supportive care; HR = hazard ratio; Bev = bevacizumab; Pem = pemetrexed*.

## Switch Maintenance

Switch maintenance is the introduction of an additional, potentially non-cross-resistant agent, immediately after completion of first-line chemotherapy in patients who achieved an objective response or a stable disease. This strategy focuses on the early integration of drugs that have been shown to be useful in the second line setting and in this regard can be seen as “early second line” therapy. This exposes the patient to new toxicity that needs to be addressed before choosing such a strategy.

The first pivotal trial to report benefit with a cytotoxic agent using this strategy was presented by Fidias et al. who randomized 309 patients with advanced NSCLC who did not progress after front-line treatment with four cycles of carbo/gemcitabine to receive immediate docetaxel maintenance therapy vs. delayed docetaxel at disease progression. The study showed a significant 3 months improvement in PFS and a non-statistically significant 2.5 months increase in OS in favor of the “immediate” docetaxel arm, with no increase in toxicity or decrease in QOL. Even though the patients in the delayed arm were carefully assessed and followed and that docetaxel was available to all of these patients, 37.2% of the patients in this arm did not receive docetaxel due to a rapid disease progression or a rapid PS decline (14).

JMEN is a phase III trial that evaluated maintenance pem vs. BSC in 633 non-progressive stage IIIB/IV patients after non-pem containing platinum-doublet chemotherapy. The pemetrexed arm showed significantly improved PFS (4.3 vs. 2.6 months; HR, 0.5, *p* < 0.0001) and OS (13.4 vs. 10.6 months; HR, 0.79; *p* = 0.012). Subgroup analysis based on histology showed that the improvement in PFS (4.5 vs. 2.6 months; HR, 0.44; *p* = 0.0001) and OS (15.5 vs. 10.3 months; HR, 0.70; *p* = 0.002) was restricted to patients with tumors having a non-squamous histology (72.5% of the population) (15). In this trial, there was a limited access to pem in the BSC arm, as only 18% of patients were treated with this drug, thus creating a subsequent imbalance in interpreting the benefit of pem as a highly active drug in this particular population.

After showing an OS benefit in second and third line setting for advanced NSCLC (16), erlotinib was evaluated as a switch maintenance therapy in SATURN, a large phase III trial that randomized 889 patients who did not progress after four cycles of a platinum doublet, to erlotinib or placebo. There was a modest but statistically significant improvement in PFS (3 vs. 2.8 months; HR 0.71, *p* < 0.0001) and OS (12 vs. 11 months; HR, 0.81, *p* = 0.0088). Subgroup analyses showed larger treatment benefit in terms of OS in patients with stable disease (HR, 0.72) after induction than in responders (HR, 0.94). Progression-free survival was significantly higher in patients with EGFR activating mutations (HR, 0.23) than in patients EGFR WT (HR, 0.78) but a survival difference could not be demonstrated in these subgroups (17). In a similar fashion to the JMEN trial, erlotinib was not widely available to patients in the placebo arm and only 21% of these patients were actually treated with erlotinib. Similar results were seen in the Erlotinib maintenance arm of the smaller IFCT-GFPC 0502 study mentioned earlier, with a 1 month improvement in PFS but no statistically significant change in OS (8). Following these positive results, Johnson et al. studied the combination of bev and erlotinib in maintenance. In the ATLAS trial, 1160 patients received first-line platinum-based chemotherapy with bev, 768 patients had an objective response or SD, and were randomized to receive bev with erlotinib vs. bev alone. This trial showed 1 month improvement in PFS (4.8 vs. 3.7 months, HR 0.72, *p* = 0.0012) for patients in the combination maintenance arm but a non-statistically significant improvement in OS (14.4 vs. 13.3 months; HR, 0.92; *p* = 0.5341) (18).

The EORTC Lung Cancer Group and the Italian Lung Cancer Project evaluated maintenance with gefitinib vs. placebo in 173 patients who did not progress after four cycles of platinum-based chemotherapy. PFS was better in the treatment arm (4.1 vs. 2.9 months; HR, 0.61; *p* = 0.0015) but with no statistically significant improvement in OS (10.9 vs. 9.4 months; HR, 0.83; *p* = 0.2) (19). The INFORM trial, an Asian phase III trial, tested gefitinib in a similar setting in 296 patients (79 EGFR-mut). PFS was significantly higher in the gefitinib arm (4.8 vs. 2.6 months; HR, 0.42; *p* < 0.0001) with more benefit EGFR-mut subgroup (HR, 0.17) compared to EGFR WT (HR, 0.87). An OS benefit was not shown (18.7 vs. 16.9 months; HR, 0.84; *p* = 0.2608) (20). Finally, the West Japan Thoracic Oncology Group 0203 phase III trial compared prolonged chemotherapy with 6 cycles of a platinum doublet to a short course of 3 cycles followed by gefitinib maintenance in 604 patients. PFS was statistically better favoring gefitinib maintenance (4.6 vs. 4.3 months; HR, 0.68; *p* < 0.001) but no significant difference in OS was found (13.7 vs. 12.9 months; HR, 0.86; *p* = 0.11) (21) (Table 2).

**Table 2:** **Key studies evaluating switch maintenance**.

Reference (study name)	*N* (pts)	Maintenance arms	PFS (mo)	HR	*p* Value	OS (mo)	HR	*p* Value
Fidias et al. (14)	309	Immediate	5.7		0.0001	12.3		0.0853
		Docetaxel						
		Delayed	2.7			9.7
		Docetaxel
Ciuleanu et al. (15)	663	Pemetrexed	4.3	0.5	<0.0001	13.4	0.79	0.012
(JMEN)		Placebo	2.6			10.6		
Cappuzzo et al. (17)	889	Erlotinib	3.0	0.71	<0.0001	12.0	0.81	0.0088
(SATURN)		Placebo	2.8			11.0	
Perol et al. (8)	310	Erlotinib	2.9	0.69	0.003	11.4	0.87	0.3043
(IFCT-GFPC 0502)		Observation	1.9			10.8	
Kabbinavar et al. (18)	768	Bev/Erlotinib	4.8	0.72	0.0012	14.4	0.92	0.5341
(ATLAS)		Bevacizumab	3.7			13.3
Gaafar et al. (19)	173	Gefitinib	4.1	0.61	0.0015	10.9	0.83	0.2
(EORTC08021-LCP01/03)		Placebo	2.9			9.4	
Zhang et al. (20)	296	Gefitinib	4.8	0.42	<0.0001	18.7	0.84	0.26
(INFORM)		Placebo	2.6			16.9	
Takeda et al. (21)	604	Gefitinib	4.6	0.68	<0.001	13.7	0.86	0.11
(WJTOG 0203)		Observation	4.3			12.9		

*PFS = progression-free survival; OS = overall survival; BSC = best supportive care; HR = hazard ratio; Bev = bevacizumab; Pem = pemetrexed*.

## Discussion

Maintenance therapy, whether in switch or continuation approach, has proved to be beneficial in patients with advanced NSCLC who have received up to four cycles of a platinum-containing regimen. Despite much debate regarding the results of the different studies and the reserved improvement in survival, Pemetrexed and Erlotinib are already approved and used for maintenance in many countries.

### Arguments against maintenance

In dealing with a population of patients with a non-curable disease, improvements in overall survival and QoL remain the primary objective. Symptomatic patients who begin induction therapy with a platinum doublet for lung cancer are often looking forward to a symptom free and drug free holiday. In this regard, many patients who obtain a meaningful symptomatic response to induction are not enthusiastic about adding on more therapy, especially if maintenance therapy is discussed after induction. Compared to continuation maintenance, switch maintenance has the added inconvenience of exposing the patient to new toxicities not encountered during induction. The interpretation of many trials is also bound with controversy. In particular, the absence of broad availability of pemetrexed in the JMEN trial or erlotinib in SATURN limits the interpretation of any small OS gain observed in these studies as these drugs are now widely available in many countries, particularly in the second line setting. The question now becomes as to whether these agents are better given before radiological or clinical progression (“early second line”) or at the time of clear progression.

### Arguments for maintenance

The current data appear even more compelling for continuation maintenance, especially for non-squamous and EGFR-mut patients. The PARAMOUNT trial has shown a PFS and an OS benefit for continuation pem in responding and stable disease patients. As used in this trial, limiting therapy to four cycles of cisplatin therapy decreases platin-related toxicities and the absence of new drug exposure limits the risk of new unexpected toxicities. Considering a median time to progression of 6–12 weeks in many observation arms of maintenance strategy trials, delaying progression is a clinically meaningful endpoint to many patients.

The case for switch maintenance is more debatable. For reasons described above, the apparent OS benefit for switching to erlotinib or pemetrexed may be associated to study design not relevant to current practice. Nonetheless, progression in the placebo arms is often very rapid as reported in trials where radiological and clinical follow up is frequent. Leaving patients without treatment can thus expose them to rapid and early progression, often leading to a decline in performance status and inability to receive further therapy. As the biggest benefit in the JMEN and SATURN trial appears to be in patients obtaining no more than SD to induction therapy, it may be hypothesized that these patients may obtain more benefit from an earlier initiation of second line therapy. As such, it seems reasonable to consider switch therapy to patients that did not obtain palliative benefit from induction therapy in an attempt to better alleviate symptoms and prevent symptomatic progression.

Some patients are identified as having an actionable mutation during induction chemotherapy. The ideal timing of the beginning of targeted therapy in this particular situation is still a matter of debate. It seems reasonable to pursue induction chemotherapy in these patients, particularly if they are responding and tolerating treatment well. On the other hand, switching to a specific targeted agent is appropriate if induction is poorly tolerated or if symptoms are poorly controlled. Targeted agents, for instance EGFR and ALK inhibitors, are associated with rapid improvement in symptoms in patients harboring sensitive mutations to these agents.

## Conclusion

Maintenance therapy has shown effectiveness in delaying progression in many studies as well as prolonging overall survival in some settings. Appropriate clinical decisions involve early discussions of these options with potentially eligible patients. Factors that may impact in the final decision to initiate maintenance include tumor histology, clinical and radiological response to induction, tumor mutations, and most importantly patient choice. Further improvements in treatment and patient selection will most likely arise with the improved refinement of the molecular diagnosis of lung cancers.

## Conflict of Interest Statement

The Guest Associate Editor Vera Hirsh declares that, despite having collaborated with author Normand Blais, the review process was handled objectively and no conflict of interest exists. The authors declare that the research was conducted in the absence of any commercial or financial relationships that could be construed as a potential conflict of interest.
